# High-frequency repetitive transcranial magnetic stimulation improves spatial episodic learning and memory performance by regulating brain plasticity in healthy rats

**DOI:** 10.3389/fnins.2022.974940

**Published:** 2022-08-05

**Authors:** Qi Wu, Xingjun Xu, Chenyuan Zhai, Zhiyong Zhao, Wenjun Dai, Tong Wang, Ying Shen

**Affiliations:** ^1^Rehabilitation Medicine Center, The First Affiliated Hospital of Nanjing Medical University, Nanjing, China; ^2^Department of Rehabilitation, Hengyang Medical School, The First Affiliated Hospital, University of South China, Hengyang, China; ^3^College of Biomedical Engineering & Instrument Science, Zhejiang University, Hangzhou, China

**Keywords:** repetitive transcranial magnetic stimulation, resting-state functional magnetic resonance imaging, cognitive improvement, neural plasticity, *N*-methyl-D-aspartic acid receptors, healthy rat

## Abstract

**Background:**

Repetitive transcranial magnetic stimulation (rTMS) is an effective way to stimulate changes in structural and functional plasticity, which is a part of learning and memory. However, to our knowledge, rTMS-induced specific activity and neural plasticity in different brain regions that affect cognition are not fully understood; nor are its mechanisms. Therefore, we aimed to investigate rTMS-induced cognition-related neural plasticity changes and their mechanisms in different brain regions.

**Methods:**

A total of 30 healthy adult rats were randomly divided into the control group and the rTMS group (*n* = 15 rats per group). The rats in the control and the rTMS group received either 4 weeks of sham or high-frequency rTMS (HF-rTMS) over the prefrontal cortex (PFC). Cognitive function was detected by Morris water maze. Functional imaging was acquired by resting-state functional magnetic resonance imaging (rs-fMRI) before and after rTMS. The protein expressions of BDNF, TrkB, p-Akt, Akt, NR1, NR2A, and NR2B in the PFC, hippocampus, and primary motor cortex (M1) were detected by Western blot following rTMS.

**Results:**

After 4 weeks of rTMS, the cognitive ability of healthy rats who underwent rTMS showed a small but significant behavioral improvement in spatial episodic learning and memory performance. Compared with the pre-rTMS or the control group, rats in the rTMS group showed increased regional homogeneity (ReHo) in multiple brain regions in the interoceptive/default mode network (DMN) and cortico-striatal-thalamic network, specifically the bilateral PFC, bilateral hippocampus, and the left M1. Western blot analyses showed that rTMS led to a significant increase in the expressions of *N*-methyl-D-aspartic acid (NMDA) receptors, including NR1, NR2A, and NR2B in the PFC, hippocampus, and M1, as well as an upregulation of BDNF, TrkB, and p-Akt in these three brain regions. In addition, the expression of NR1 in these three brain regions correlated with rTMS-induced cognitive improvement.

**Conclusion:**

Overall, these data suggested that HF-rTMS can enhance cognitive performance through modulation of NMDA receptor-dependent brain plasticity.

## Introduction

Alzheimer’s disease is the main cause of dementia and is quickly becoming one of the most expensive, lethal, and burdensome diseases of this century. In 2018, Alzheimer’s Disease International estimated the prevalence of dementia at about 50 million people worldwide, a number projected to triple by 2050, with two-thirds of those with the disease living in low-income and middle-income countries (Scheltens et al., 2021). Although there is no consensus yet on the origin of AD, one of the dominant working hypotheses is involved in the progressive deficits in neural plasticity resulting from the amyloid-β (Aβ) cascade (Battaglia et al., 2007; Li et al., 2021). A healthy nervous system is endowed with synaptic plasticity and other neural plasticity, which are believed to be key physiological mechanisms for learning and memory. There has been much anatomical and functional evidence pointing toward AD as a kind of disconnection syndrome, which is manifested as a decline in the linkages between different brain areas in the cognitive network, and neural plasticity dysfunction (Brier et al., 2014). However, few available medications have successfully reversed or affected the disease course of AD. Because it affects neural plasticity both at the site of stimulation and in remote brain regions and acts through functional anatomical connections, rTMS has been regarded as a potentially safe and cost-effective treatment for AD (Li et al., 2021). Understanding the potential mechanisms of rTMS for cognitive improvement of brain plasticity has been meaningful for preventing and treating AD.

Resting-state functional magnetic resonance imaging has been shown to disrupt a distributed network, especially the linkages between the hippocampus, the prefrontal cortex (PFC), and other brain areas, pathologically involved in preclinical AD (Zarei et al., 2013). Ample evidence has demonstrated that overall brain plasticity declines in AD patients (Battaglia et al., 2007; Cai et al., 2017; Noguchi-Shinohara et al., 2021). Plasticity in the PFC, hippocampus, and several other brain regions was impaired in both patients with AD and APP/PS1 mice (Battaglia et al., 2007). Plasticity in the number and strength of neural connections is the physical basis of learning and memory (Todd et al., 2019). Therefore, the enhancement of brain activity and the triggering of specific structural and functional changes promoted by neural plasticity effects could be expected to improve cognitive abilities (Li et al., 2014). The use of rTMS could induce temporary excitation/inhibition and long-term effects in specific cortical areas as well as in deep sites via the magnetic field, the effects of which can last for a considerable time. Over recent years, rTMS was broadly applied when investigating the changes across cortical networks (Stagg et al., 2010; Kohl et al., 2019). Previous evidence has demonstrated that the dorsolateral prefrontal cortex (DLPFC), the core part of PFC, plays crucial roles in various cognitive tasks such as working memory, episodic memory, attention, problem-solving, etc. (Giglia et al., 2021; Jones and Graff-Radford, 2021). Abnormal activities of the DLPFC as well as functional disconnection involving DLPFC have been observed in MCI and AD (Cai et al., 2017). Considering its local dysfunction and roles in various neural circuits relevant to the physiological mechanisms of cognitive impairment, the function of the DLPFC and its relationship with the other brain regions in AD or MCI should be especially emphasized. HF-rTMS over the DLPFC can improve cognitive function in patients with MCI and AD (Chou et al., 2020). Previous studies have shown that rTMS over the DLPFC modified cognitive performances via modulating brain functional connectivity of DLPFC and other brain regions in MCI or AD patients (Guo et al., 2021; Esposito et al., 2022). Thus, rTMS-induced activity and neural plasticity underlie cognitive improvement. However, to our knowledge, the specific activity and neural plasticity in different brain regions that affect cognition by rTMS over the PFC or DLPFC are not fully understood.

High-frequency rTMS can induce long-lasting PFC-hippocampus neural plasticity and improve cognitive function (Ma et al., 2014; Li et al., 2019). However, it is not clear how rTMS impacts neural plasticity in different brain regions. The NMDA receptors play a central role in synaptic plasticity, which is closely related to learning and memory abilities (Gribkova and Gillette, 2021). Functional NMDA receptors are tetramers composed of different subunits (NR1, NR2A-D, NR3A-B). The NR1, NR2A, and NR2B subunits have been demonstrated to be essential for the regulation of synaptic plasticity in the adult hippocampus (Tsien et al., 1996). NMDA receptor dysfunction has been reported to play a role in the pathophysiology of AD (Lin et al., 2014). A 10-Hz rTMS produced a long-term potentiation-like excitatory effect through NMDA receptor-dependent glutamatergic activity (Brown et al., 2021). Zhang et al. (2015) reported that rTMS facilitated spatial cognition and synaptic plasticity associated with increasing levels of BDNF and NMDA receptors. To date, researchers have mainly focused on synaptic plasticity of the hippocampus, and few studies have examined synaptic plasticity-related intracellular signaling pathways in different brain regions responding to rTMS. The mechanism of rTMS on NMDA receptors of distant sites in the brain is still unclear.

It has been shown that rTMS can improve various cognitive domains in several disease models. However, although its exploration in healthy animals is essential to attribute its pure effect in learning and memory processes, there have been few studies in this regard. Addressing healthy animals contributes to a better understanding of the basic mechanisms by which rTMS improves cognitive function and its modulation (Zorzo et al., 2021). The understanding of how rTMS promotes cognitive effects in healthy animals is crucial for directing the power of rTMS to reduce the burden of cognitive decline. Also, it is important to know how rTMS and its mechanisms affect neural plasticity in different brain regions in healthy rats. In the present study, we explored the cognitive improvement effect of rTMS on healthy rats, focusing on cognitive-related neural plasticity changes and their underlying mechanisms in different brain regions. We believe it would be noteworthy for preventing and treating neural plasticity impairment-related diseases.

## Materials and methods

### Ethics statement

All experimental procedures and protocols were approved by the Ethics Committee of Nanjing Medical University (Animal Ethics Number: 2007005-2) and were designed to minimize the suffering and number of animals used. All the procedures were conducted with strict attention to safe animal care and use following National Institutes of Health guidelines for humane animal care.

### Animals and study design

**FIGURE 1:**
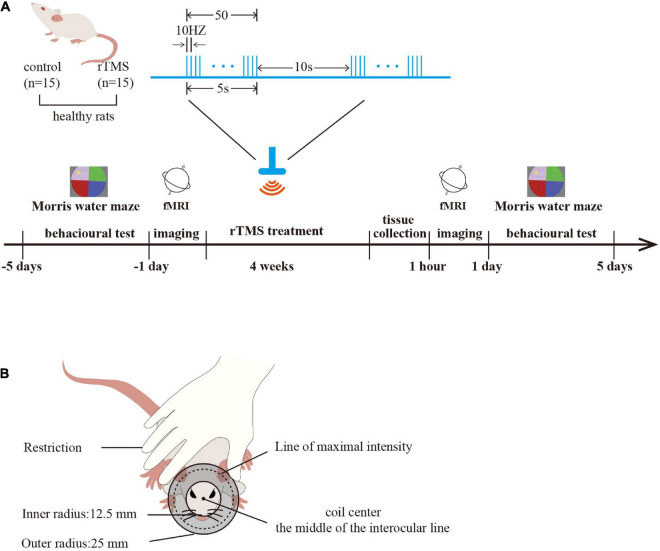
Study protocol. **(A)** Experimental timeline and parameters of one rTMS session. Timeline showing a time series event of rat treatment. Healthy rats received rTMS intervention (rTMS: real, rTMS group; sham, control group) for 4 weeks. The brain tissues were harvested 1 h after rTMS intervention (*n* = 6 rats per group), and the spatial cognition function was tested in the Morris water maze (MWM) before and after rTMS intervention (*n* = 6 rats per group). rs-fMRI was used to detect the activation of the brain before and after rTMS intervention (*n* = 3 rats per group). One rTMS session consisted of 10 burst trains, with each train containing 10 pulses at 10 Hz with 10-s intertrain intervals. **(B)** A schematic diagram of rat stimulation. The circular coil was positioned over the rat’s head and the coil center was placed over the middle of the interocular line with the handle pointing forward.

A total of 30 male adult Sprague Dawley rats (250–300 g) were included in the study. All the rats were provided by the Experimental Animal Center of Nanjing Medical University (Medical Experimental Animal Number: SYXK2018-20020). The rats were randomly divided into a control group and an rTMS group (*n* = 15 rats per group). For each group of experiments, the animals were matched by age and body weight. All the rats were housed in animal facilities under controlled conditions (55 ± 5% relative humidity, 24 ± 2°C, and a 12/12 h light/dark cycle). Animals were allowed to acclimate to these conditions for at least 7 days before inclusion in the experiments. Rats in the rTMS group received 4 weeks of HF-rTMS intervention, and rats in the control group received sham rTMS intervention. Each group had six animals for behavioral testing, six for Western blot analysis, and three for rs-fMRI. After 4 weeks of intervention, rats for molecular testing in each group were sacrificed with pentobarbital (50 mg/kg, intraperitoneal) and the brain tissues, including the PFC, hippocampus, and M1, were harvested (Figure 1A).

### Repetitive transcranial magnetic stimulation protocol

Rats in the rTMS group engaged in corresponding real rTMS intervention 5 days per week for four consecutive weeks in the awake state. The coil was turned by 90° and placed 5 cm away from the skull for the sham rTMS in the control group to ensure auditory conditions were similar but did not receive brain stimulation. The CCY-IA rTMS apparatus was supplied by Wuhan Yiruide Medical Equipment Co., LTD (Wuhan, Hubei, China). Repetitive TMS was implemented with a parallel-wound solenoidal circular coil stimulator (Y064, height = 20.4 mm, 50-mm outer diameter, 25-mm inner diameter, number of turns = 6 layers × 5 turns/layer = 30 turns) with 3.5-T peak magnetic welds, specifically designed for rodents. Rats were hand-restrained in a suitable cloth when treated with rTMS. The coil center was placed over the middle of the interocular line with the handle pointing forward (Gersner et al., 2011). One rTMS session consisted of 10 burst trains, with each train containing 10 pulses at 10 Hz with 10-s intertrain intervals, totaling 1,000 pulses, lasting 10 min. The motor threshold (MT) intensities when using rats were measured preliminarily (Gersner et al., 2011). The stimulation intensity was set at 80% of the average MT of rats. In our study, the mean of MT intensities (*n* = 6 per group) of rats was 42.60 ± 9.10% of the maximum stimulator output (Figures 1A,B).

### Functional magnetic resonance imaging acquisition

Data on fMRI were acquired on a 7.0-T MRI scanner (Biospec 7T/20 USR, Bruker Biospin, Ettlingen, Germany). A quadrature volume resonator (inner diameter of 72 mm) was used for radiofrequency transmission, and a four-element surface coil array was used for signal reception. The rats (*n* = 3 rats per group) were anesthetized with 2% isoflurane and NO_2_/O_2_ mixed gas anesthesia in a chamber. During the MRI scan, the rat was prostrated on a custom-made holder to minimize head motion. An echo-planar imaging (EPI) sequence was used with the following scanning parameters: matrix size = 256 × 256, flip angle = 70°, resolution = 0.5 mm × 0.5 mm, slice thickness = 1 mm, slice gap = 0, repetition time (TR) = 330 ms, echo time (TE) = 10 ms, slice number = 25.

### Functional magnetic resonance imaging data analysis

Image preprocessing of the rs-fMRI data was performed for all individuals using the SPM12^1^ toolkit, which included the following steps: (1) Slice time correction: correcting the time information of each layer of each volume of each rat to eliminate the phase difference of the time series between layers; (2) Head motion correction: the first volume of each rat was used as a reference standard, and the remaining volumes were spatially registered to the referred image using a 6-parameter rigid-body transformation to eliminate head movements. At the same time, the averaged image after head motion correction was generated; (3) Spatial normalization: a template was used as a reference standard, and the averaged image after head motion correction was used as the source image to estimate the registration parameters (12-parameter affine transformation and non-linear deformation), and then applied to normalize the brain images of all the tested rats to the template space to eliminate the differences among the rats. (4) Nuisance signals regression: 13 parameters (six head movement + six head movement first derivative + whole-brain signal) were regressed; (5) Detrend: removing device linear signal drift; (6) Filtering: the filter is 0.01–0.1 Hz. After that, regional homogeneity (ReHo) was used to measure the “coherence” of local brain activity. The principle is to calculate the Kendall harmony coefficient between each pixel and its surrounding 12 pixels. Then, Gaussian smoothing and Z-transformation were performed for ReHo maps. For each state of all rats before and after treatment, the calculated ReHo index was subjected to a single-sample test (the threshold was *p* < 0.001) and FDR correction, and then the statistics of each brain function index were generated under the threshold of cluster size > 50 to analyze the results. The paired *t*-test was performed for each brain function index before and after treatment in all rats; the threshold was uncorrected *p* < 0.05, cluster size = 50.

### Spatial episodic learning and memory test by using Morris water maze

Morris water maze (MWM) tests were used to evaluate the spatial learning and memory 1 day before and 1 day after rTMS treatment in rats from each group (*n* = 6 rats/group), as previously described (Hong et al., 2020). Morris water labyrinth equipment (JLBehv-MWMG, Shanghai Jiliang Technology Co., Ltd., Shanghai, China) included a circular pool and a video photography tracking system. The pool (1.5-m diameter and 0.5-m in height) was filled with 22–24°C black water. A hidden platform (9 cm in diameter) that served as the escape platform was submerged 2.0 cm below the water surface. The rat faced the wall of the pool and was gently placed in the water. The first four consecutive days constituted the latency trial when the rats were trained to find the escape platform once a day. The average time required to complete the task was called “full average escape latency.” Rats were considered to have achieved it if they remained on the platform for 2 s. If rats failed to reach the platform within 60 s, they would be manually guided to the platform and held there for 15 s to familiarize themselves with the environment and the platform position before being placed back into the cage. At this time, the escape latency was recorded as 60 s. The latency and swimming path for reaching the platform were recorded for each rat, and its decline over days of training reflected learning and memory. On the fifth day, the platform was removed to perform a probe trial, and each rat was allowed to search the maze for 60 s. The rats were placed into the quadrant opposite the first quadrant and the number of platform crossings within the 60 s was recorded. This exploratory test was used to assess the memory level and spatial cognitive ability. The escape latency to find the platform, the time spent in the target quadrant, and platform crossings were tracked and analyzed by the ANY-maze video tracking software (Stoelting, Keele, WI, United States).

### Western blot analyses

Tissue samples of the PFC, hippocampus, and M1 were dissected and homogenized in RIPA lysis buffer (Beyotime, Shanghai, China). Total protein was quantified by a BCA assay (Beyotime, Shanghai, China), separated by SDS-PAGE, and transferred to PVDF membranes (Millipore, Boston, MA, United States). The primary antibodies were as follows: rabbit anti-BDNF (ab108319, Abcam, Cambridge, Cambridgeshire, Umnited States), rabbit anti-TrkB (ab179515, Abcam, Cambridge, Cambridgeshire, UK), rabbit anti-Akt (T55972, Abmart, Shanghai, China), rabbit anti-p-Akt (4060S, Cell signal technology, Boston, MA, United States), rabbit anti-NR1 (5704S, Cell signal technology, Boston, MA, United States), rabbit anti-NR2A (ab124913, Abcam, Cambridge, Cambridgeshire, United States), rabbit anti-NR2B (4207S, Cell signal technology, Boston, MA, United States), and rabbit anti-β-actin (wx488142, ABclonal, Wuhan, Hubei, China). After rinsing, the membranes were incubated with HRP-conjugated secondary antibodies. The densities of the bands on the membranes were visualized by enhanced chemiluminescence (ChemiScope6100, Qinxiang, Shanghai, China), followed by exposure to X-ray film (RX-U, Fujifilm, Tokyo, Japan). Finally, the results were quantified by using NIH Image J software (Bethesda, MD, United States) and expressed as a ratio to the β-actin protein.

### Statistical analyses

Statistical analyses were performed using GraphPad Prism software (version 9.0 c, GraphPad Software, Inc., La Jolla, CA, United States) and SPSS (version 21, SPSS Inc., IBM, Armonk, NY, United States). A Kolmogorov–Smirnov test showed that all data were normally distributed. Data with normal distributions are expressed as the mean ± SEM. Two-way ANOVA with Tukey’s test for multiple comparisons was used to determine differences among individual groups. The unpaired *t*-test was used when comparing two separate groups. Spearman’s correlation was used to analyze the correlation between the relative band intensity of NR1 with the performance in the MWM task. For all statistical significance, the levels were set at *P* < 0.05.

## Results

### Use of repetitive transcranial magnetic stimulation over the prefrontal cortex improved spatial episodic learning and memory abilities in healthy rats

**FIGURE 2:**
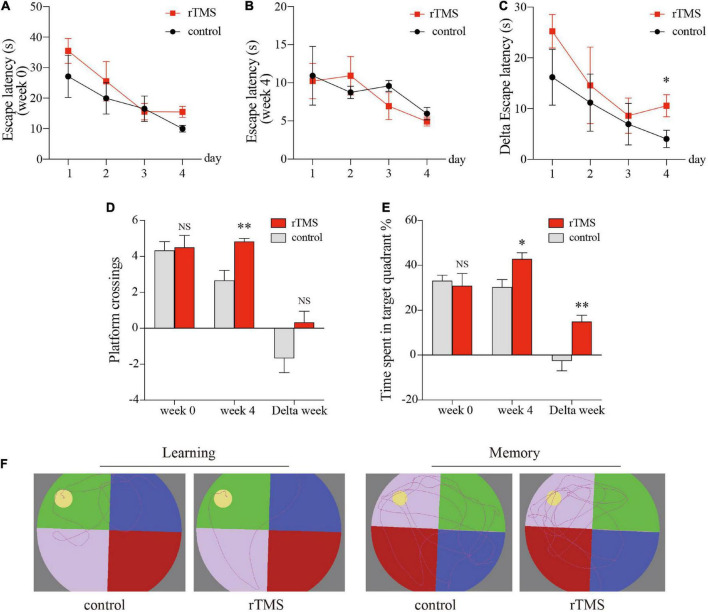
rTMS over the PFC improved spatial episodic learning and memory ability in healthy rats. At 4 weeks post rTMS, rats in indicated groups were applied for Morris water maze (MWM) tests. Latencies of pre- and post-rTMS (**A,B**, respectively), delta latencies of pre- and post-rTMS **(C)**. **(D)** The frequency of swimming across the platform location in the probe trial. **(E)** Percentage of time spent in the platform quadrant in the probe trial. **(F)** Representative track plots of rats in hidden platform test (left plots, “learning”) and probe trial stage (right plots, “memory”). Data are shown as mean ± SEM; *n* = 6 per group; *P < 0.05 and ^**^P < 0.01 vs. control group.

We used the MWM test to evaluate the effect of rTMS on spatial episodic learning and memory in healthy rats. In the spatial learning stage, all rats from different groups benefited from the 4-day training and exhibited gradually decreased latency to the platform (Figures 2A–C). In the latency trial, there were no significant differences in the escape latencies between the control group and the rTMS group in week 0 (Figure 2A) and week 4 (Figure 2B), while the delta latency of pre- and post-rTMS showed that the escape latencies of rTMS group decreased more significantly after 4 weeks of rTMS than the control group (rTMS vs. control, two-way ANOVA, *F* = 4.405, *P* < 0.05 for time, *F* = 39.097, *P* < 0.0001 for group, *F* = 0.035, *P* > 0.05 for day × group Figure 2C). In the spatial probe trial, cognitive performance was represented by the frequency of swimming across the platform and the percentage of time spent in the platform quadrant (Figures 2D,E). The frequency of swimming across the platform in week 4 in the rTMS rats was small but significantly higher than in the control rats (*P* < 0.05, Figure 2D). The change in percentage of time spent in the target quadrant in week 4 and the delta time spent in the target quadrant was significantly higher in the rTMS group than in the control group (*P* < 0.05 and *P* < 0.01, respectively, Figure 2E). These results proved that rTMS treatment could enhance spatial episodic learning and memory ability in healthy rats.

### Use of repetitive transcranial magnetic stimulation over the prefrontal cortex increased regional homogeneity in the prefrontal cortex, hippocampus, primary motor cortex, and other brain regions in healthy rats

**FIGURE 3:**
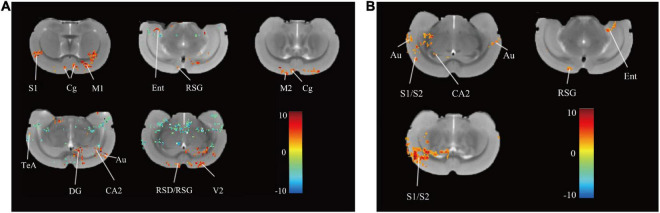
Differences in ReHo before and after rTMS and between group. **(A)** Brain regions with significant differences in ReHo between pre- and post-rTMS in the rTMS group (*P* < 0.05, uncorrected, cluster size = 50). The hot color represents the higher ReHo in post-rTMS. The cold color represents the higher ReHo in pre-rTMS. **(B)** Brain regions with significant differences in ReHo between the rTMS and the control group after rTMS (*P* < 0.05, uncorrected, cluster size = 50). The hot color represents the higher ReHo in the rTMS group. S1, primary somatosensory cortex; Cg, cingulate cortex; M1, primary motor cortex; Ent, medial entorhinal cortex; RSG, retrosplenial granular cortex; M2, secondary motor cortex; RSD, retrosplenial dysgranular cortex; TeA, temporal association cortex; V2, secondary visual cortex; DG, dentate gyrus; CA2, cornus ammonis 2; Au, auditory cortex; S2, secondary somatosensory cortex.

After rTMS intervention, 13 regions in the rTMS group, namely, the right primary somatosensory cortex (S1), left and right cingulate cortex (Cg), left primary motor cortex (M1), right medial entorhinal cortex (Ent), right retrosplenial granular cortex (RSG), right secondary motor cortex (M2), right retrosplenial dysgranular cortex (RSD), right temporal association cortex (TeA), left dentate gyrus (DG), left cornus ammonis 2 (CA2), left auditory cortex (Au), and left secondary visual cortex (V2) showed increased ReHo (*P* < 0.05, uncorrected, cluster size = 50, Figure 3A) over pre-rTMS. The rTMS group also showed increased ReHo in the left and right Au, right primary/secondary somatosensory cortex (S1/S2), right CA2, right RSG, and left Ent as compared to the control group (*P* < 0.05, uncorrected, cluster size = 50, Figure 3B). Previous studies reported that structures in the frontal module (orbital cortex, prelimbic cortex, cingulate cortex 1, and cingulate cortex 2) belong to the architectonic subdivision of the “orbital medial prefrontal cortex” (OPFC) (Hsu et al., 2016; Carlén, 2017), and DG and CA 2 belong to the hippocampus. In addition, PFC (Cg), hippocampus (DG and CA2), M1/M2, and TeA are the brain regions important for the interoceptive/default mode network (DMN), whereas the Ent, RSD, and S1/S2 belong to the cortico-striatal-thalamic network. Hippocampus, RSG, and Au are important brain regions of both the interoceptive/DMN and cortico-striatal-thalamic network. Therefore, use of rTMS over the PFC could activate the interoceptive/DMN and cortico-striatal-thalamic network in healthy rats.

### Use of repetitive transcranial magnetic stimulation over the prefrontal cortex increased the protein expressions of *N*-methyl-D-aspartic acid receptors and activated the brain-derived neurotrophic factor/TrkB/Akt pathway in the prefrontal cortex, primary motor cortex, and hippocampus in healthy rats

**FIGURE 4:**
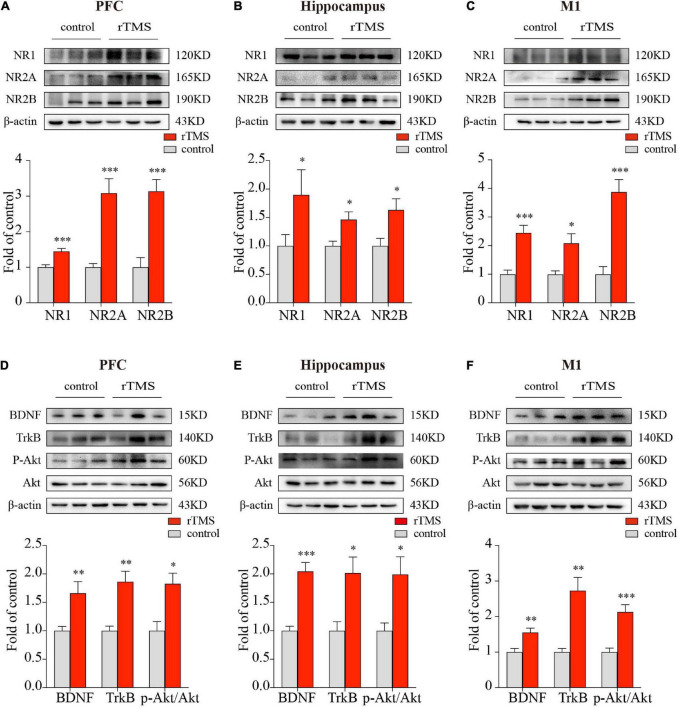
Western blot analysis of synaptic plasticity-related proteins in the PFC, hippocampus, and M1. The representative blots depict NMDA receptors compositions in brain tissues from PFC **(A)**, hippocampus **(B)**, as well as M1 **(C)**. The representative blots depict the BDNF/TrkB/Akt pathway compositions in brain tissues from PFC **(D)**, hippocampus **(E)**, as well as M1 **(F)**. Data are shown as mean ± SEM; *n* = 6 per group; *P < 0.05, ^**^P < 0.01, and ^***^P < 0.001 vs. control group.

Since NMDA receptors play a central role in synaptic plasticity, the promotion of synaptic plasticity depends on the synthesis of new plasticity-related proteins. To further evaluate the effect and mechanisms of rTMS on brain plasticity, we examined the synaptic plasticity-related proteins NMDA receptors and their interaction with the BDNF/TrkB/Akt pathway through the use of Western blot. We detected the protein expressions of NR1, NR2A, and NR2B in the PFC, hippocampus, and M1. In the PFC, hippocampus, and M1, rTMS over the PFC significantly increased NR1(*P* < 0.001, Figure 4A; *P* < 0.001, Figure 4B; *P* < 0.001, Figure 4C, respectively), NR2A (*P* < 0.001, Figure 4A; *P* < 0.01, Figure 4B; *P* < 0.001, Figure 4C, respectively), and NR2B (*P* < 0.001, Figure 4A; *P* < 0.05, Figure 4B; *P* < 0.001, Figure 4C, respectively). To explore the mechanism by which rTMS upregulates NMDA receptors, we focused on BDNF, which stands out for its high level of expression in the brain and its potent effects on synapses. We found rTMS increased the protein expressions of BDNF in the PFC, hippocampus, and M1 (*P* < 0.01, Figure 4D; *P* < 0.001, Figure 4E; *P* < 0.01, Figure 4F, respectively). Moreover, we examined TrkB, the receptor of BDNF, and its downstream p-Akt and Akt in these three brain regions. Western blot showed rTMS increased TrkB (*P* < 0.01, Figure 4D; *P* < 0.05, Figure 4E; *P* < 0.01, Figure 4F, respectively) and p-Akt/Akt (*P* < 0.05, Figure 4D; *P* < 0.05, Figure 4E; *P* < 0.001, Figure 4F, respectively) in the PFC, hippocampus, and M1. All these results indicated that in the PFC, hippocampus, and M1, rTMS-induced neural plasticity was, at least partly, NMDA receptor-dependent synaptic plasticity, and rTMS-induced increase of NMDA receptors might depend on activation of the BDNF/TrkB/Akt pathway in the PFC, hippocampus, and M1.

### The synaptic plasticity-related protein NR1 changes in the prefrontal cortex, hippocampus, and primary motor cortex correlated to a cognition improvement in the repetitive transcranial magnetic stimulation-treated healthy rats

**FIGURE 5:**
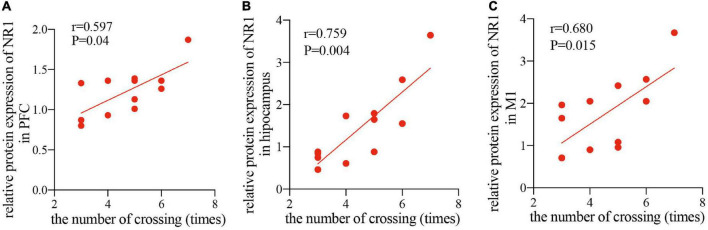
Correlation scatters plot of the number of times crossing and the relative protein expression of NR1 in healthy rats after rTMS. Correlation between the number of crossings in the MWM task and the relative protein expression of NR1 in the PFC **(A)** (*r* = 0.597, *P* < 0.05), hippocampus **(B)** (*r* = 0.759, *P* < 0.01), and M1 **(C)** (*r* = 0.659, *P* < 0.05).

Since the NR1 subunit is considered indispensable for functional NMDA receptor assemblies, whereas, NR2A and NR2B subunits are not present at all synaptic NMDA receptors (Papouin et al., 2012), therefore, to study the relationship between the improved cognitive function by rTMS and the NMDA receptor-dependent neural plasticity, we conducted a correlation analysis between the relative band intensity of NR1 and performance in the MWM task (number of crossings) in the PFC, hippocampus, and M1. The analysis showed that after 4 weeks of rTMS, the number of times crossing the platform, reported in the water maze exploration experiment, was positively correlated with the relative protein expression of NR1 in healthy rats in the PFC (*r* = 0.597, *P* < 0.05, Figure 5A), hippocampus (*r* = 0.759, *P* < 0.01, Figure 5B), and M1 (*r* = 0.659, *P* < 0.05, Figure 5C). Taken together, rTMS-induced improvement of cognitive function was significantly correlated with the expression of NMDA receptors in the PFC, hippocampus, and M1.

## Discussion

Although a huge number of studies has drawn upon patients or animals with cognitive impairment to study the effects of rTMS on neural plasticity, especially in the PFC and hippocampus, relatively few cognitive impairment studies have focused on the effects of rTMS on the neural plasticity of brain regions other than the PFC and hippocampus, especially the M1 region. Moreover, the potential mechanisms of the enhanced plasticity benefits of rTMS in the PFC for cognitive improvement remain unclear. In this study, we evaluated the ReHo values of the brain using rs-fMRI and detected synaptic plasticity-related proteins to reflect neural plasticity. This study demonstrated that 4 weeks of rTMS over the PFC improved spatial episodic learning and memory ability and promoted brain plasticity via activating the interoceptive/DMN and cortico-striatal-thalamic network in healthy rats, and in the PFC, hippocampus, and M1, rTMS-induced NMDA receptor-dependent synaptic plasticity correlated to the cognitive improvement. Furthermore, we found rTMS-induced synaptic plasticity in these brain regions was accompanied by activation of the BDNF/TrkB/Akt/NMDAR pathway. We first provide evidence that rTMS-induced cognitive enhancement-related neural plasticity in both the delivered site and the distant sites are, at least partly, a result of NMDA receptor-dependent synaptic plasticity, and the mechanism of the neural plasticity in these activated brain regions might be the activation of the BDNF/TrkB/Akt pathway.

### Use of repetitive transcranial magnetic stimulation over the prefrontal cortex improves spatial episodic learning and memory

Repetitive TMS has been shown to improve cognitive impairment in AD patients (Chou et al., 2020; Li et al., 2021). We further investigated the effect of rTMS on cognitive function in healthy rats. The MWM test was conducted after 4 weeks of real/sham rTMS on healthy rats to evaluate their cognitive behaviors. Previous studies suggest that there are many options for intervention sites for rTMS. The PFC, especially the DLPFC, is a core region involved in cognitive functioning, such as working memory and executive functions, and DLPFC plasticity is correlated with cognitive ability in AD (Balderston et al., 2020; Chou et al., 2020; Jones and Graff-Radford, 2021). Therefore, the PFC was chosen as the intervention site in this study. Solé-Padullés et al. (2006) first represented the evidence that rTMS over the left DLPFC is capable of transitorily and positively influencing brain function and cognition among the elderly with memory complaints. A recent systematic review and meta-analysis showed that high-frequency rTMS over the left DLPFC and low-frequency rTMS on the right DLPFC improved memory functions in MCI and AD (Chou et al., 2020). Moreover, several meta-analyses comparing the TMS-induced effect of the different region’s stimulation protocols (i.e., left DLPFC) in AD or MCI, showed that the benefits induced by rTMS are most likely due to the stimulation of the left DLPFC (Begemann et al., 2020; Chou et al., 2020). These findings support our use of HF-rTMS over the PFC to investigate cognitive enhancement in healthy rats. Our results showed that rTMS over the PFC could improve spatial learning and memory in healthy rats (Figure 2). Consistent with our study, Zhang et al. (2015) and Shang et al. (2019) found HF-rTMS can enhance spatial episodic learning and memory in cognitively impaired animal models, which may be related to the improvement of neural plasticity in the brain.

### Use of repetitive transcranial magnetic stimulation over the prefrontal cortex increases regional homogeneity in brain regions in interoceptive/default mode network and the cortico-striatal-thalamic network

Neural plasticity has been defined in terms of the capacity to acquire cognitive skills (Carlén, 2017). It is therefore not surprising that neural plasticity abnormalities are present in patients with cognitive impairment (Burke and Barnes, 2006). The blood-oxygenation-level dependent (BOLD)-fMRI approach has long been used to assess brain plasticity. In this study, brain activity was measured synchronically by using ReHo during rs-fMRI, which showed that rTMS over the PFC increased PFC activity, manifested by the increased ReHo in the PFC, including the left and right cingulate cortex (Figure 3A), which was consistent with Yin et al. (2020).

Functional imaging studies indicate that rTMS can affect the physiological functions of not only the brain area below its coil but also relatively distant brain areas. Increasing evidence shows that spatial learning and memory loss are a severe consequence of neural plasticity disturbance in the hippocampus. The two main areas that form the hippocampus – the cornus ammonis 1, 2, and 3 (CA1, CA2, and CA3) and the dentate gyrus (DG) – contribute separately to spatial learning and memory processes. Converging evidence has suggested that DLPFC-hippocampus network connectivity is pathologically involved in preclinical AD (Goveas et al., 2011). In parallel, dialog between the hippocampus and the PFC allows the memory to reemerge into consciousness, which promotes the consolidation and storage of episodic memories (Preston and Eichenbaum, 2013). In this study, although the delivered target of rTMS was the PFC, after rTMS, the ReHo in the rTMS group also increased in CA2, DG, and other brain regions as compared to the pre-rTMS or control group (Figures 3A,B). These results suggested that rTMS over the PFC directly induced the neural plasticity in the PFC, and representations in the hippocampus were boosted by indirect manipulation. Consistent with our study, Wang et al. (2014, 2018) found that the targeted enhancement of PFC-hippocampal brain networks improved associative semantic and episodic memory performance, which involved localized long-term plasticity. The roles of the hippocampus and PFC in memory processing, individually or in concert, are a major topic of interest in memory research, and PFC and hippocampus are strongly connected by direct and indirect pathways (Eichenbaum, 2017). Therefore, rTMS over the PFC improves spatial episodic learning and memory via promoting neural plasticity in the PFC-hippocampus.

In recent years, AD was regarded as a disease associated with the deterioration of neural plasticity in the whole brain. Altered functional connectivity between the DLPFC, posterior hippocampus, and other brain regions with advanced age may contribute to age-related differences in episodic memory (Ankudowich et al., 2019). Therefore, targeting rTMS at the DLPFC may promote interconnected network activity and integration, which may be directly related to the multi-domain cognitive improvements observed (Alcalá-Lozano et al., 2018). Previous studies demonstrated that the DLPFC is considered a key region contributing to several large-scale brain networks, such as the interoceptive/DMN, FPN, and CEN (Liston et al., 2014; Sarpal et al., 2020; Zheng et al., 2020). In this study, compared with both the pre-intervention (Figure 3A) and the control groups (Figure 3B), the brain regions activated after rTMS were parts of the interoceptive/DMN or cortico-striatal-thalamic network. We also found some reports that were consistent with our results. Dunlop et al. (2016) reported that rTMS over the dorsomedial PFC successfully modulated cortico-striatal connectivity in obsessive-compulsive disorder. By using [^18^F]-FDG microPET, Parthoens et al. (2014) found that rTMS over the PFC of rats increased neural activity in the medial entorhinal cortex, which is involved in spatial learning and memory (Ga et al., 2013). Hippocampal-targeted theta-burst stimulation enhanced associative memory formation via modulating parahippocampal and retrosplenial cortices (Tambini et al., 2018), which are involved in episodic-like and spatial memory (Sato, 2021). Koch et al. (2018) reported that rTMS enhanced memory and neural activity in AD patients by manipulating DMN. These all support the fact that rTMS over the PFC could improve spatial learning and memory and promote neural plasticity by activating interoceptive/DMN and the cortico-striatal-thalamic network, which is consistent with our findings.

### Repetitive transcranial magnetic stimulation-induced neural plasticity is, at least partly, *N*-methyl-D-aspartic acid receptor-dependent synaptic plasticity

Resting state-fMRI in this study provided indirect evidence that HF-rTMS over the PFC increased neural plasticity in the PFC, hippocampus, M1, and other brain regions, which belong to the interoceptive/DMN and the cortico-striatal-thalamic network. A previous study showed that stimulating this region directly excited neurons, and consequently reduced the synaptic conduction threshold, thereby making the synapse become relatively active and increasing the synaptic connections (Gribkova and Gillette, 2021). Several basic studies reported that HF-rTMS improved cognitive function by regulating synaptic plasticity in rodents with cognitive impairment (Zhang et al., 2015; Shang et al., 2019). Therefore, as a form of neural plasticity, synaptic plasticity needs concern in this study.

*N*-Methyl-D-aspartic acid receptors, the major mediator of postsynaptic response during synaptic neurotransmission, are thought to be able to generate a persistent increase in synaptic strength and participate in the regulation of synaptic plasticity, memory, and cognition. Activation of NMDA receptors allows a calcium flux into dendritic spines that serve as the proximal trigger for synaptic plasticity. Impaired NMDA receptors’ function is regarded as the most likely cause of the NMDA receptor-dependent cortical plasticity deficit in aging and AD (Battaglia et al., 2007). Using GLYX-13, an NMDA receptor glycine site functional partial agonist, could enhance cognition (Moskal et al., 2014). Whereas, the NMDA receptor antagonist memantine was regarded as a symptomatological and neuroprotective treatment for AD (Danysz and Parsons, 2003). Therefore, we detected the protein expression of NR1, NR2A, and NR2B in several brain regions. Consistent with the result of the rs-fMRI, we also found rTMS significantly increased synaptic plasticity in the PFC, hippocampus, and M1, as manifested by the increased protein expression of synaptic plasticity-related proteins NR1, NR2A, and NR2B in these brain regions, indicating rTMS-induced neural plasticity might be synaptic plasticity (Figures 4A–C). Consistent with our results, Zhang et al. (2015) and Shang et al. (2016), respectively, found that rTMS facilitated spatial cognition and synaptic plasticity associated with up-regulation of NR1, NR2A, and NR2B in the hippocampus. Based on the above, the rTMS-induced NMDA receptor-dependent synaptic plasticity occurred not just locally in the targeted delivered site but also in the non-stimulated brain regions at distant sites, and it is reasonable to believe that NMDA receptor-dependent synaptic plasticity is, at least partly, the underlying mechanism of rTMS-induced neural plasticity.

### Use of repetitive transcranial magnetic stimulation over the prefrontal cortex induces upregulation of *N*-methyl-D-aspartic acid receptors by activating the brain-derived neurotrophic factor/TrkB/Akt pathway

Another concern is how rTMS upregulates NMDA receptors in different brain regions. It is now widely accepted that the main function of BDNF in the adult brain is to regulate synapses, with structural and functional effects ranging from short-term to long-lasting, on excitatory or inhibitory synapses, in many brain regions. Previous studies showed that rTMS improved cognition, increased BDNF-NMDAR pathway level and synaptic plasticity, and enhanced cognitive behavior in cognitively impaired patients or animal models (Zhang et al., 2015; Velioglu et al., 2021). The effects of BDNF in rTMS-induced synaptic plasticity are mediated by its specific membrane-bound receptor TrkB (tropomyosin-related kinase B) receptors (Shang et al., 2019). A previous study showed that increasing the phosphorylation of Akt in hippocampal neurons by activation of BDNF/TrkB signaling would upregulate NMDA receptors and promote synaptic plasticity (Nakai et al., 2014), and cognitive decline in different models accompanied by decreasing the level of BDNF/TrkB signaling (Mercerón-Martínez et al., 2021). In addition, Li et al. (2022) reported that activation of Akt promoted the interaction of cAMP-response element-binding protein (CREB) and the promoter of NR2B to increase NR2B. Kay et al. (2013) and Hu et al. (2015), respectively, reported that activation of Akt/CREB also increased NR1 and NR2A, and that the inhibition of BDNF-PI3K/Akt or Akt/CREB pathways led to chronic nervous system impairment. Thus, we speculated that rTMS increased NMDA receptors via activating BDNF/TrkB/Akt pathway. The increase of NMDA receptors accompanied by increasing in BDNF, TrkB, and p-Akt/Akt in the PFC, hippocampus, and M1 verified the speculation (Figures 4D–F). Therefore, rTMS promotes NMDA receptor-dependent synaptic plasticity by activating BDNF/TrkB/Akt pathways in the PFC, hippocampus, and M1.

### Repetitive transcranial magnetic stimulation-induced upregulation of NR1 in the prefrontal cortex, hippocampus, and primary motor cortex correlates with cognitive enhancement

A number of studies have shown that rTMS-induced neural plasticity in the PFC or hippocampus correlated with cognition enhancement (Wang et al., 2014, 2018). Recently, Li et al. (2019) reported that 6 weeks of HF-rTMS treatment over the left DLPFC improved long-term potentiation-like plasticity in M1 in AD patients, and the long-term potentiation-like plasticity improvement in M1 correlated to the observed cognition change. Considering that no other studies to date have reported cognition-related plasticity was shown in the M1 after rTMS over the PFC, therefore, based on Li’s study, in addition to the PFC and hippocampus, we also found that rTMS over the PFC led to an increase in ReHo in the M1 by rs-fMRI and an upregulation of synaptic plasticity-related proteins by Western blot in M1, indicating rTMS-induced NMDA receptor-dependent synaptic plasticity in the PFC, hippocampus, and M1. The NR1 subunit is often considered indispensable for functional NMDA receptor assemblies, thus we detected the correlation between protein expressions of NR1 and rTMS-induced cognitive changes. More specifically, we found protein expressions of NR1 in the PFC, hippocampus, and M1 correlated with rTMS-induced cognitive changes (Figures 5A–C). In clinical practice, M1 plasticity is relatively easy to acquire. Therefore, in the future, detection of the neural plasticity in the M1, and perhaps in other brain regions, might be a potential assessment tool reflecting cognition and the therapeutic effect of rTMS. Collectively, enhancement of the cognitive abilities by rTMS over the PFC is associated with modulation of the neural plasticity of interoceptive/DMN and cortico-striatal-thalamic network in the brain.

## Limitations

Several limitations are inherent in the present study. First, we only demonstrated that the PFC rTMS can improve cognition in healthy rats, but we have not verified it in disease models. Whether the effects of rTMS observed in the healthy rats could also be replicated in cognitively impaired rats should be investigated further. Second, our sample size, especially in terms of the number of rats who underwent rs-fMRI, was small, thus the functional connectivity analysis-related study could not be performed in this study. Finally, stimulation from the coil could not be accurately localized to a specific brain area because of the small size of the rat brain compared with the size of the coil. Therefore, we could not ensure that each stimulation was applied to the exact same area. Further study will be required to rigorously evaluate whether effects are truly inverted and if the same inversion is present for other stimulation targets.

## Conclusion

The present results demonstrated that HF-rTMS over the PFC improved spatial episodic learning and memory and promoted brain plasticity via activating interoceptive/DMN and cortico-striatal-thalamic network in healthy rats. In addition, rTMS-induced brain plasticity, locally in the targeted delivered site and in the non-stimulated regions at distant sites, was NMDA receptor-dependent synaptic plasticity, which was achieved through activating the BDNF/TrkB/Akt pathway. In conclusion, we demonstrated that HF-rTMS can enhance cognitive performance through the modulation of NMDA receptor-dependent brain plasticity.

## Data availability statement

The original contributions presented in this study are included in the article/supplementary material, further inquiries can be directed to the corresponding authors.

## Ethics statement

The animal study was reviewed and approved by the Ethics Committee of Nanjing Medical University.

## Author contributions

YS, QW, and XX contributed to the conception and design of the study. XX and QW performed the experiments. QW, ZZ, and CZ conducted the statistical analyses. TW and ZZ provided expert interpretation of the data. QW and YS drafted the manuscript. WD, QW, and YS edited and confirmed the final manuscript. YS and TW were the principal investigators of the study and were responsible for the study conception and interpretation of the data, and had final responsibility for the decision to submit the manuscript for publication. All authors listed have made a substantial, direct, and intellectual contribution to the work, and approved it for publication.

## Footnotes

1
http://www.fil.ion.ucl.ac.uk/spm/

## Abbreviations

AD: Alzheimer’s disease
MCI: mild cognitive impairment
rTMS: repetitive transcranial magnetic stimulation
PFC: prefrontal lobe
HF-rTMS: high-frequency repetitive transcranial magnetic stimulation
rs-fMRI: resting-state functional magnetic resonance imaging
FC: functional connectivity
OPFC: orbital medial prefrontal cortex
DMN: default mode network
FPN: fronto-parietal network
CEN: central executive network
BDNF: brain-derived neurotrophic factor
NMDA: *N*-methyl -D-aspartic acid
S1: primary somatosensory cortex
Cg: cingulate cortex
M1: primary motor cortex
M2: secondary motor cortex
Ent: medial entorhinal cortex
RSG: retrosplenial granular cortex
RSD: retrosplenial dysgranular cortex
V2: secondary visual cortex
DG: dentate gyrus
CA2: cornus ammonis 2
Au: auditory cortex
TeA: temporal association cortex
S2: secondary somatosensory cortex.
